# Impacts of Cardiovascular Health and Hypertensive Disorders of Pregnancy on Early-Midlife Cardiac Structure and Function

**DOI:** 10.1016/j.jacadv.2026.103045

**Published:** 2026-07-22

**Authors:** Jane M. Wei, Janet M. Catov, Kathryn L. Berlacher, Flordeliza S. Villanueva, Malamo E. Countouris

**Affiliations:** a Division of General Internal Medicine, Department of Medicine, University of Pittsburgh Medical Center, Pittsburgh, Pennsylvania, USA; b Department of Obstetrics, Gynecology, and Reproductive Sciences, University of Pittsburgh, Pittsburgh, Pennsylvania, USA; c Department of Epidemiology, University of Pittsburgh, Pittsburgh, Pennsylvania, USA; d Division of Cardiology, Department of Medicine, University of Pittsburgh Medical Center, Pittsburgh, Pennsylvania, USA

**Keywords:** CVH, echocardiography, hypertensive disorders of pregnancy, preeclampsia, remodeling

**What is the clinical question being addressed?**

How do CVH and HDP history impact cardiac structure and function a decade post-delivery?

**What is the clinical question being addressed?**

HDP is associated with worse CVH a decade after delivery, yet some associations with adverse measures of LV remodeling and diastolic dysfunction persist even after accounting for CVH. Those with HDP and low CVH had the most significant differences in echocardiographic parameters, representing an opportunity for CVH factor modification.

Hypertensive disorders of pregnancy, including gestational hypertension and preeclampsia, are associated with increased risks of adverse cardiovascular outcomes.^1^ Subclinical left ventricular (LV) remodeling can be seen by echocardiography a decade after delivery, most pronounced in those with preeclampsia history who develop hypertension.^2^ The cardiovascular health (CVH) score, developed from the American Heart Association’s “Life’s Essential 8,” includes 4 health factors (body mass index [BMI], total cholesterol, blood pressure, and insulin resistance) and 4 health behaviors (smoking, physical activity, sleep, and diet)^3^ that may affect the risk of subclinical cardiac changes after hypertensive disorder of pregnancy (HDP). Established cardiovascular risk factors mediate some of the association between HDP and overt cardiovascular disease.^1^ However, despite calls to optimize CVH following HDP, there is no clear evidence on how composite CVH influences subclinical cardiovascular measures following HDP. This exploratory study aims to investigate the association between HDP history and CVH, allowing for examination of how early-midlife CVH may impact the effects of HDP history on echocardiographic measures of cardiac structure and function.

Individuals who delivered between 2008 and 2009 were identified from the Magee Obstetric Maternal and Infant database at the University of Pittsburgh 7 to 15 years after delivery.^4^ Of these, a subset without prepregnancy clinical cardiovascular disease or hypertension (n = 233) returned for an echocardiogram study visit at which they underwent 2-dimensional transthoracic B-mode and Doppler echocardiography, had blood pressure and BMI measured, completed a comprehensive survey about medical history, medications, and health behaviors, and underwent a lab draw. HDP was assessed through chart review, and echocardiographic measurements were obtained by a blinded sonographer and read by 2 blinded cardiologists, as previously described.^2^ Diastolic dysfunction was assessed according to 2025 American Society of Echocardiography/European Association of Cardiovascular Imaging (ASE/EACVI) guidelines. As left atrial reservoir strain was not recorded, individuals who may have met diastolic dysfunction criteria with this parameter were marked as inconclusive. In accordance with the American Heart Association’s “Life’s Essential 8” Cardiovascular Health Score,^3^ data covering 8 domains were collected, and each converted to its respective component of CVH into a score from 1 to 100, which was averaged to obtain a total CVH score out of 100.^5^ CVH was further categorized (low <50, moderate ≥50 and <80, and high ≥80).^3^ Given the small number of individuals with HDP history and high CVH (n = 2), moderate and high CVH were combined for subgroup analyses. Linear and logistic regression analyses investigated contributions of demographic variables, HDP status, and CVH score on continuous and categorical echo measures, respectively. Subgroups defined by both HDP history and CVH category (HDP/low CVH, HDP/moderate-high CVH, no HDP/low CVH, and no HDP/moderate-high CVH) were compared using analysis of variance (ANOVA)/Kruskal-Wallis for continuous variables, chi-squared/Fisher’s exact tests for categorical variables, and post hoc tests with Bonferroni pairwise comparisons. The study received approval by the University of Pittsburgh Institutional Review Board (STUDY19110290).

Of 233 individuals (mean age 38 ± 6 years, follow-up 11 ± 1 years from index delivery, 34% self-identified as Black), 52 (22%) had HDP (19 gestational hypertension and 33 preeclampsia) during the index pregnancy. Those with HDP were more likely to be primiparous at index pregnancy (69% vs 51%, *P* value = 0.031). In those without HDP in the index pregnancy, 4% had preeclampsia in a subsequent pregnancy. Those with HDP had higher BMI (34.7 ± 7.4 vs 29.8 ± 7.7 kg/m^2^), rates of stage 1 or greater hypertension (56% vs 25%), blood pressures (systolic blood pressure 123 ± 11 vs 114 ± 13, diastolic blood pressure 81 ± 9 vs 74 ± 10 mm Hg), and lower CVH (58 ± 13 vs 65 ± 12), all *P* < 0.001; lifestyle behaviors were similar, although a minority in the overall cohort had ideal sleep (39%), diet (9%), and physical activity (11%). In regression models adjusted for CVH, age, education level, and gravidity, those with HDP had a thicker interventricular septum (IVS) (β 0.062 cm, 95% CI: 0.01-0.11 cm, *P* = 0.019) and lower E/A (β −0.157, 95% CI: −0.28 to −0.03, *P* = 0.013). In analyses by HDP history and CVH category (Table 1), there were differences across subgroups for IVS, LV posterior wall, diastolic dysfunction proportion, E/A, e’, and left atrial volume index (LAVI) (all *P* < 0.05). In pairwise comparisons, those with HDP/low CVH had thicker IVS (1.08 ± 0.18 vs 0.91 ± 0.15 cm, *P* = 0.001) and LV posterior wall (1.06 ± 0.20 vs 0.90 ± 0.13 cm, *P* < 0.001), higher prevalence of diastolic dysfunction (9% vs 0%, *P* = 0.01) with lower E/A (1.04 ± 0.30 vs 1.37 ± 0.39, *P* = 0.024) and average e’ (9.3 ± 1.6 vs 11.5 ± 2.1 cm/s, *P* = 0.003), and lower LAVI (18.2 ± 7.2 vs 25.0 ± 6.7 mL/m^2^, *P* = 0.023) when compared with no HDP/moderate-high CVH.

**Table 1:** Echocardiographic Variables by HDP/CVH Subgroups

	No HDP/Mod-High CVH (n = 142)	HDP/Mod-High CVH (n = 38)	No HDP/Low CVH (n = 26)	HDP/Low CVH (n = 11)	*P* Value
Ejection fraction (%)	63.2 ± 5.5	63.8 ± 6.0	61.3 ± 4.6	64.7 ± 5.5	0.241
LVEDd (cm)	4.5 ± 0.5	4.7 ± 0.5	4.7 ± 0.4	4.5 ± 0.5	0.067
IVS thickness (cm)	0.91 ± 0.15	0.97 ± 0.19	0.97 ± 0.16	1.08 ± 0.18^g^	0.001
LVPW thickness (cm)	0.90 ± 0.13	0.94 ± 0.14	0.97 ± 0.14	1.06 ± 0.20^g^	<0.001
LAVI (mL/m^2^)	25.0 ± 6.7	24.5 ± 7.7	22.8 ± 7.9	18.2 ± 7.2^g^	0.023
LVMI (g/m^2^)	73.0 ± 15.5	76.7 ± 17.1	78.4 ± 16.1	77.6 ± 18.5	0.268
Relative wall thickness^a^	0.40 ± 0.07	0.41 ± 0.09	0.41 ± 0.08	0.49 ± 0.13	0.086
LV remodeling, n (%)^b^	51 (35.9)	13 (34.2)	11 (42.3)	8 (72.7)	0.098
LV hypertrophy, n (%)^c^					0.310
No hypertrophy	80 (58.0)	21 (56.8)	14 (53.9)	3 (27.3)	
Concentric remodeling	46 (33.3)	11 (29.7)	9 (34.6)	7 (63.6)	
Eccentric hypertrophy	9 (6.5)	3 (8.1)	1 (3.9)	0 (0)	
Concentric hypertrophy	3 (2.2)	2 (5.4)	2 (7.7)	1 (9.1)	
E/A ratio	1.37 ± 0.39	1.27 ± 0.37	1.31 ± 0.29	1.04 ± 0.30^g^	0.024
Septal e' (cm/s)^a^	10.1 ± 2.1	10.1 ± 2.9	9.6 ± 3.5	8.0 ± 1.6^g^^,^^h^	0.013
Lateral e' (cm/s)	12.9 ± 2.8	12.4 ± 2.9	11.1 ± 2.5^g^	10.7 ± 2.0	0.002
Average e’ (cm/s)	11.5 ± 2.1	11.3 ± 2.6	10.3 ± 2.5	9.3 ± 1.6^g^	0.003
E/e' ratio^a^	7.2 ± 1.7	7.8 ± 2.0	8.1 ± 2.2	7.2 ± 2.7	0.079
Diastolic function, n (%)^d^					0.011
No diastolic dysfunction	127 (89.4)	31 (81.6)	26 (100)	8 (72.7)	
Diastolic dysfunction	0 (0)	2 (5.3)	0 (0)	1 (9.1)	
Inconclusive	15 (10.6)	5 (13.2)	0 (0)	2 (18.2)	
Reduced e’, n (%)^e^	1 (0.7)	4 (10.5)	3 (11.5)	1 (9.1)	0.002
Peak TR velocity (m/s)^f^	2.0 ± 0.3	2.0 ± 0.3	1.9 ± 0.4	2.0 ± 0.2	0.545

Values are mean ± SD unless otherwise indicated.

CVH = cardiovascular health; HDP = hypertensive disorder of pregnancy; IVS = interventricular septum; LAVI = left atrial volume index; LV = left ventricular; LVEDd = left ventricular end diastolic diameter; LVMI = left ventricular mass index; LVPW = left ventricular posterior wall; TR = tricuspid regurgitant; RWT = relative wall thickness.

a Kruskal-Wallis performed given equal variances and normality assumptions not met.

b LV remodeling is RWT>0.42.

c No hypertrophy is LVMI≤95 g/m^2^, RWT<0.42; concentric remodeling is LVMI≤95 g/m^2^, RWT>0.42; eccentric hypertrophy is LVMI>95 g/m^2^, RWT≤0.42; concentric hypertrophy is LVMI>95 g/m^2^, RWT>0.42.

d Diastolic dysfunction was a composite of diastolic parameters, based on 2025 American Society of Echocardiography/European Association of Cardiovascular Imaging (ASE/EACVI) guidelines, excluding left atrial reservoir strain as this was not available.

e Reduced e’ was average e’≤6.5, septal e’≤6, or lateral e’≤7 cm/s.

f Peak TR velocity was only available for 71% of the population given lack of measurable TR jet in remaining participants.

g Significantly different from no HDP/mod-high CVH.

h Significantly different from HDP/mod-high CVH.

In this cohort of early-midlife individuals followed a decade postpartum, those with HDP history had lower CVH, driven by CVH factors (particularly hypertension and BMI), rather than behaviors. Notably, only 4% of individuals with HDP history had high (ideal) CVH. HDP was associated with measures of LV wall thickness and diastolic parameters, even when adjusting for CVH. When stratified by HDP and CVH subgroups, those with HDP history and subsequent low CVH had the most adverse differences in echocardiographic parameters, including the highest LV wall thickness, most adverse diastolic function parameters, and the highest rate of diastolic dysfunction.

To our knowledge, this is the first study to consider CVH, a composite risk score incorporating both biological and behavioral factors, and its impact on the association of HDP with subclinical structural and functional cardiac changes. A key inference from our study is that just 1 decade postpartum, women with HDP had lower CVH, with 96% of those with HDP having a low or moderate CVH health score and thus potentially benefitting from targeted prevention. In our cohort, CVH factors of hypertension and BMI differed by HDP status, whereas CVH behaviors did not; however, small numbers limit subgroup analyses. Identifying the specific drivers of subclinical changes in cardiac structure and function following HDP is important, given that these are early-midlife women (average age 38 years) who are at a critical point for targeted cardiovascular disease prevention strategies to avoid later-in-life disease. Our results suggest that despite contributing factors of hypertension, there may be mechanisms unique to HDP (eg, elevated Activin A, microvascular dysfunction^5^), that impact subclinical cardiac changes. Thus, studies investigating effective cardiovascular disease prevention interventions in midlife, not just immediately postpartum, are needed.^1^

This study has some limitations. Due to small numbers in the low CVH/HDP subgroup and modest effect sizes, it may be underpowered for subgroup conclusions, and thus our findings are exploratory. Diastolic dysfunction may be underestimated, as we did not have left atrial reservoir strain measurements by echo. Our findings of smaller LAVI among those with HDP were discordant with other echo measures. Limited conclusions can be made regarding this as we only assessed atrial size, not function. CVH was collected at a single time point in early-midlife; thus, conclusions about temporality are limited. Lastly, there may be an element of selection bias, as only a subset of eligible women underwent echocardiography.

In this cohort of early-midlife individuals a decade after delivery, those with HDP had significantly lower CVH, likely driven by higher blood pressure and BMI. Yet, HDP status was independently associated with IVS thickness, an LV remodeling measure, and E/A ratio, a diastolic dysfunction parameter, even when adjusted for CVH, suggesting there are likely other drivers of LV remodeling after HDP that warrant further investigation. These exploratory analyses suggest that those with HDP history and low CVH have the most significant changes in LV structure and diastolic measures, although larger studies are needed to replicate these findings. This highlights the importance of interventions to improve CVH after HDP.

## Funding support and author disclosures

This research was funded in part by the Pittsburgh Innovation in Collaborative Training of Residents Alliance (R38 HL150207), an AHA Go Red for Women Grant (16SFRN28930000), and 10.13039/100000968AHA CDA 941351. Data storage through an institutional grant, Clinical and Translational Science Institute at the 10.13039/100007125University of Pittsburgh Grant (UL1-TR-001857). The authors have reported that they have no relationships relevant to the contents of this paper to disclose.
